# Study of a Si-based light initiated multi-gate semiconductor switch for high temperatures

**DOI:** 10.1038/s41598-022-19767-4

**Published:** 2022-09-15

**Authors:** Chongbiao Luan, Hongwei Liu, Jiabin Fu, Yang He, Le Xu, Lingyun Wang, Jianqiang Yuan, Longfei Xiao, Zhuoyun Feng, Yupeng Huang

**Affiliations:** 1 Key Laboratory of Pulsed Power, Institute of Fluid Physics, China Academy of Engineering Physics, P.O. Box 919-108, Mianyang, 621900 China; 2 Institute of Noval Semiconduction, Shandong University, Jinan, 250100 China; 3 State Key Laboratory of Crystal Materials, Shandong University, Jinan, 250100 China

**Keywords:** Electrical and electronic engineering, Optoelectronic devices and components

## Abstract

Light initiated multi-gate semiconductor switch (LIMS) is a kind of power electronic device which has many differences from traditional thyristor triggered by electric pulse. LIMS is triggered by laser, the turn-on time is smaller, and the anti-electromagnetic interferences is strong. The opening mode of LIMS is obviously different to traditional thyristor. After the laser into the gate area, a large number of electrons and holes will appear in P-base region, holes gather in the area of P-base in PN junction J2, and electrons gather in N-drift region around the PN junction J2. PN junction J2 will open first, then PN junction J3 opens. The delay time of the NPN and PNP thyristors is close to zero when the laser pulse is narrow and the peak power is high, so the turn-on velocity is fast. To optimize the characteristics of the LIMS at high temperatures, we propose a new structure of the LIMS with the optimization of the n^+^ layer, circular light gate, and the new-style edge termination. The diameter of the LIMS is 23 mm. The experiment results show that the leakage current of the proposed LIMS has been decreased from more than 1 mA to 500 μA at 125 °C, the output current of the LIMS is 10.2 kA with a voltage of 4 kV at 85 °C, and the output current of the LIMS is 12.1 kA with a voltage of 4 kV at − 55 °C. Additionally, di/dt is larger than 30 kA/μs.

## Introduction

As the most powerful semiconductor switches, electrically-triggered and light-triggered thyristors are the devices of choice for ultra-high voltage power applications, such as high voltage direct current (HVDC) transmission or pulsed power application^1–5^. Compared with the electrically-triggered thyristor, the light-triggered thyristor has more advantages in simplifying the driver circuit and improving electromagnetic compatibility^6^. However, in the ultra-high pulsed power system, such as for railgun applications, the turn-on time is short and the di/dt of the thyristor is high, resulting in traditional electrically-triggered and light-triggered thyristors being unable to fulfill the above requirement. Accordingly, the light initiated multi-gate semiconductor switch (LIMS) has been proposed. LIMS is a kind of power electronic device that possesses many differences from the traditional thyristors triggered by electrical or light pulses. The LIMS is triggered by lasers, and the turn-on velocity is fast, where the di/dt is higher than 60 kA/μs.

However, the structure of the LIMS is similar to the thyristor, which contains four layers of different doping, forming an N-P-N and a P-N-P bipolar transistor. Under high operating temperatures, the leakage current of the LIMS will increase amplified by the transistor gains, leading to the parasitic turn-on of the thyristor. This will degrade the functioning of the application in some cases, such as for military, utility, and aerospace applications^6,7^.

The study shows that the thyristor leakage current at high temperatures can also come from surface currents at the chip terminal^8^. Then, adequate edge termination and passivation techniques are necessary to minimize the leakage current, representing a significant part of the total leakage current in the LIMS.

In this paper, a new structure of the LIMS with the optimization of the n^+^ layer, circular gate, and the new-style edge termination has been proposed, with the diameter of the LIMS being 23 mm. The leakage current of the proposed LIMS is about 500 μA at 125 °C, and the output current of the LIMS is 10.2 kA with a voltage of 4 kV at 85 °C.

## Principle of LIMS

**Figure 1:**
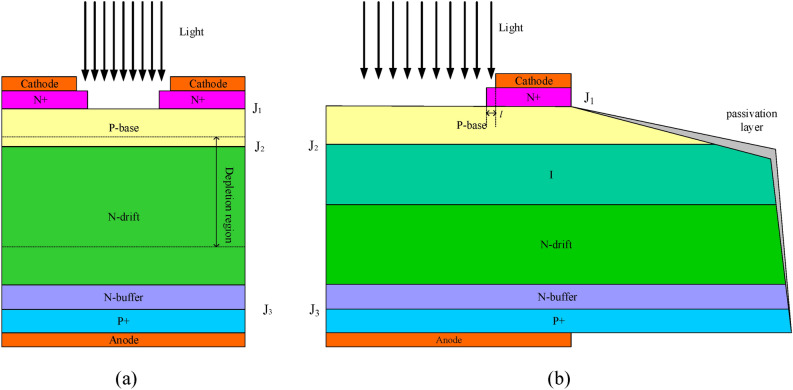
Structure of the traditional Si LIMS chip (**a**), and the optimizational Si LIMS chip (**b**).

Figure 1a is the structure of the traditional Si LIMS chip. The LIMS is almost identical to the structure of thyristor, except for the gate area, to excite more carriers by photons. However, the opening mode of the LIMS is obviously different from the traditional electrically triggered thyristor. After the laser is pointed into the light-triggered area, many electrons and holes will appear in the P-base region, holes gather around P-base in the PN junction *J*_*2*_, and electrons gather in the N-drift region around the PN junction *J*_*2*_. When the laser pulse is narrow and the peak power is high, the NPN thyristor will open before the PNP thyristor, but the delay time of the NPN and PNP thyristors is minimal. When the laser energy in unit time (laser energy and pulse width) is suitable, the PNP thyristor and NPN thyristor will open at one time. Therefore, the turn-on velocity of the LIMS is fast.

## Characteristics of LIMS at high temperatures

The current I of the LIMS can be expressed by the following equation^9^

1 $${\text{I(t)}} = \frac{{{\text{I}}_{{\text{O}}} }}{{\alpha_{{{\text{pnp}}}} }}(e^{{\frac{t}{{\sqrt {t_{npn} t_{pnp} } }}}} - 1)$$

Here, *I*_*o*_ is the light-triggered current, and *t*_*npn*_ and *t*_*pnp*_ are the transportation times of the carriers at the p-base and n-drift area, respectively.

2 $$t_{npn} = \frac{{W_{p}^{2} }}{{2D_{n} }},t_{pnp} = \frac{{(W_{n} - W_{dn} )^{2} }}{{2D_{p} }}$$

where *W*_*p*_ and *W*_*n*_ are the thicknesses of p-base and n-drift, respectively; *W*_*dn*_ is the thickness of the depletion layer; and *D*_*n*_ and *D*_*p*_ are the diffusion coefficients of the electron and hole, respectively.

The di/dt of the LIMS can be expressed by the following equation^9^:

3 $$\frac{dI(t)}{{dt}} = \frac{{{\text{I}}_{{\text{O}}} }}{{\alpha_{{{\text{pnp}}}} \sqrt {t_{npn} t_{pnp} } }}e^{{\frac{t}{{\sqrt {t_{npn} t_{pnp} } }}}}$$

The di/dt is related to the light-triggered current, the thickness of p-base, n-drift, and the depletion layer.

The key leakage current of the LIMS can be expressed by the following equation^10^:

4 $$I_{l} = \frac{{qD_{p} n_{i}^{2} }}{{L_{p} N_{D} }} + \frac{{qn_{i} W_{dn} }}{2\tau }$$

Here, *L*_*p*_ is the diffusion length of the hole, *N*_*D*_ is the dopant concentration of the n-drift, *n*_*i*_ is the intrinsic carrier concentration, and *τ* is the carrier lifetime. It can be seen that, from Eqs. (3) and (4), the leakage current and di/dt will be affected by *W*_*dn*_ and *D*_*p*_. *L*_*p*_, *N*_*D*_, and *τ* will affect the leakage current, and *D*_*p*_, *L*_*p*_, *N*_*D*_, and *τ* are all related to the temperature *T*. Therefore, the leakage current and di/dt at high temperature should be considered comprehensively while setting parameters of the LIMS chip.

Based on the above theory, the leakage current and turn-on velocity (di/dt) are related to the thickness of the depletion layer, a new structure of the LIMS with the optimization of the n^+^ layer, and circular gate, so the new-style edge termination has been proposed^11^. To decrease the leakage current at high temperatures, the n^−^ (i) layer has been inserted between the p-base and n-drift, and the passivation layer (SiO_2_) has been added at the beveled termination, which will decrease the surface state density.

**Figure 2:**
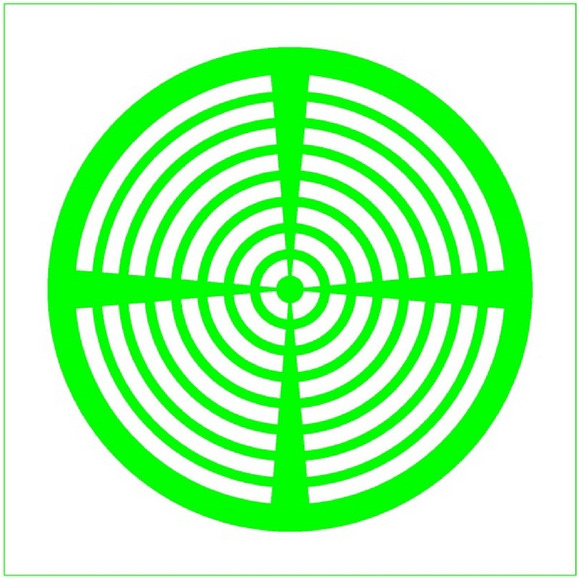
Electrode structure of cathode for the LIMS (the green area is the cathode electrode, and the other is the light triggered area).

To increase the turn-on velocity (di/dt), a multi-light triggered electrode structure of cathode for LIMS has been proposed, as shown in Figs. 1b and 2. The study shows that the distance *l* (Fig. 1b) of the n^+^ layer extends from the cathode electrode edge to the light triggered area, affecting the current’s peak value when the di/dt is high. Accordingly, the optimization of the n^+^ layer is important to the characteristics of the LIMS.

## Simulation

The LIMS was modeled with Sentaurus. In the simulation, the structure, as is shown in Fig. 1b, was modeled in 2D dimensions. For the optimizational LIMS, the thickness and dopant concentration of the n^+^ layer are 10 μm and 1 × 10^20^ cm^−3^, respectively. The thickness and dopant concentration of the p-base layer are 35 μm and 2 × 10^17^ cm^−3^, respectively. The thickness and dopant concentration of the n^−^ layer are 100 μm and 4 × 10^12^ cm^−3^, while those for the n-drift layer are 800 μm and 1.2 × 10^13^ cm^−3^, respectively. Furthermore, the thickness and dopant concentration of the p^+^ layer are 15 μm and 6 × 10^17^ cm^−3^, respectively. The distance *l* (Fig. 1b) of the n^+^ layer extending from the cathode electrode edge to the light triggered area is about 30 μm. Also, the passivation layer (SiO_2_) has been added at the beveled termination.

Additionally, the anode current was simulated by solving model equations in cylindrical coordinates. The optical window and cathode electrode are shown in Fig. 2. The monochromatic optical source was set to be uniformly irradiated to the optical windows.

**Figure 3:**
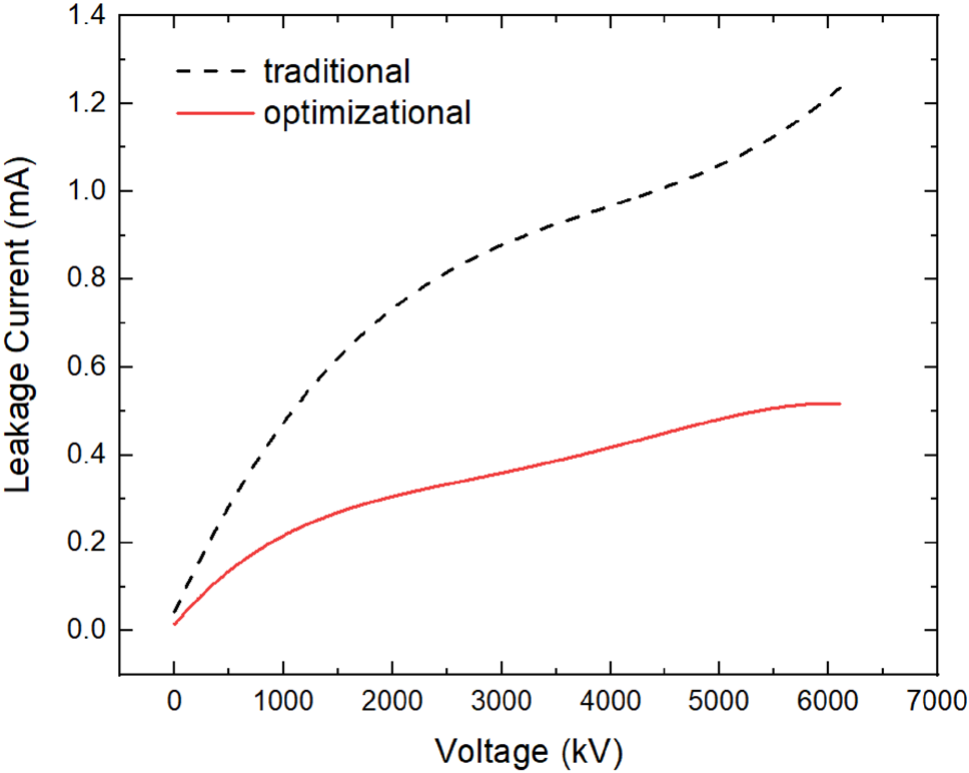
Simulation result of leakage current for the traditional and the optimizational LIMS.

Figure 3 presents the simulation results. The simulation results in Fig. 3 show that the leakage current of the LIMS with the optimization of the n^+^ layer, circular gate, and the new-style edge termination is 480 μA with a dc voltage of 5 kV and a temperature of 125 °C (the leakage current of the traditional thyristor in Fig. 1a is larger than 1 mA, as shown in Fig. 3).

## Experiment and results

**Figure 4:**
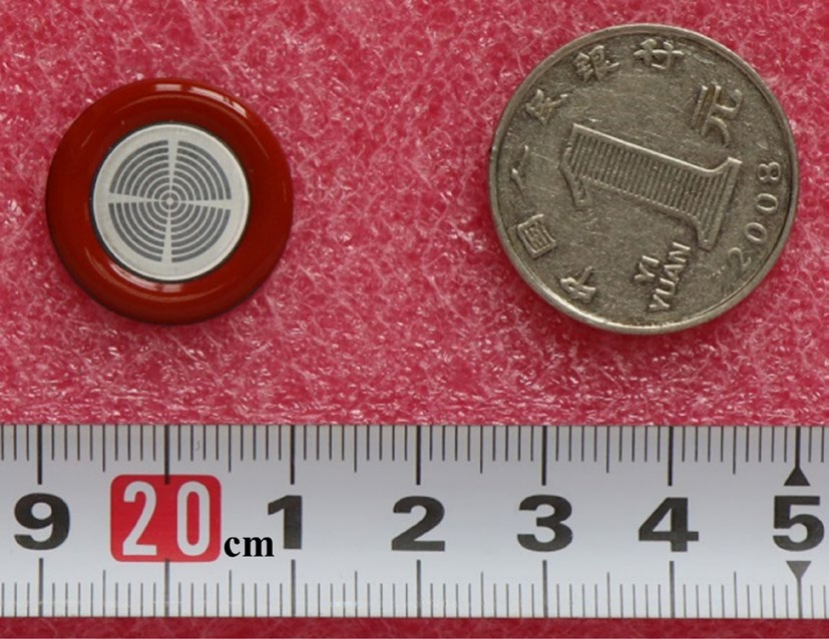
Picture of the Si LIMS chip.

The Si LIMS used in this paper was in a n^+^pn^−^np^+^ structure, with the schematic representation shown in Fig. 1b. The thickness and dopant concentration of the n^+^ layer, p-base layer, n^−^ layer, n-drift layer, p^+^ layer for the LIMS are the same as the description in the section "Simulation". The distance *l* of the n^+^ layer extending from the cathode electrode edge to the light triggered area is about 30 μm. Figure 4 presents the picture of the prepared Si LIMS, with the diameter of the LIMS chip being 23 mm.

**Figure 5:**
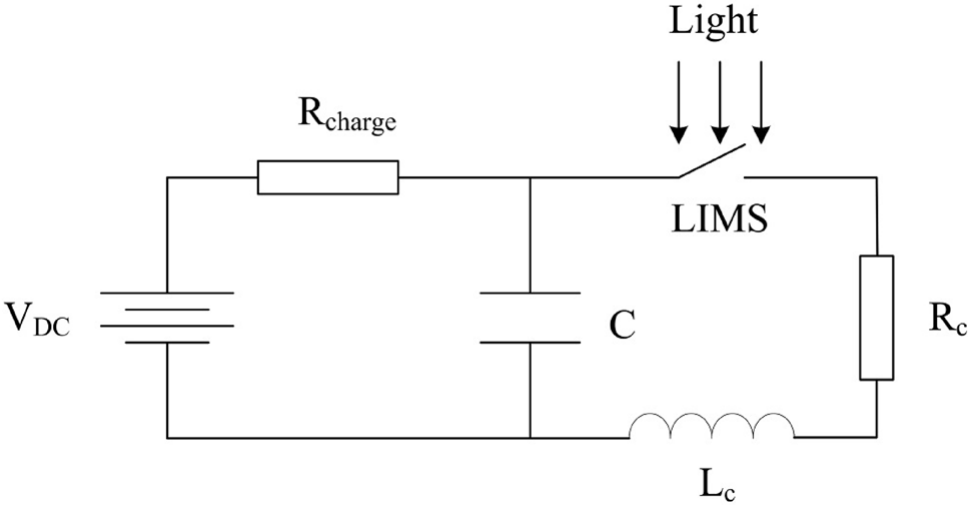
Schematic circuit diagram for evaluating the switching characteristics of the LIMS.

Figure 5 is the schematic circuit diagram for evaluating the switching characteristics of the LIMS. In the figure, *C* represents the storage capacitor (1 μF), *R*_*charge*_ is the charging resistor (1 kΩ), *Rc* is the load resistor, and *Lc* is the stray inductance. Herein, *Rc* corresponds to the resistance of LIMS and Load. *Lc* is the parasitic inductance, which originates from the layout wiring of the LIMS and other devices. A 980 nm LD (with the laser energy of 120 μJ and pulse width of 200 ns) was used as a light source to trigger the Si LIMS. The output current measurements through the LIMS were performed using a Rogowski coil whose sensitivity and response time were 0.1 V/A and 2.2 ns, respectively. The input voltage measurement was measured with the Tektronix P6015A High Voltage Probe.

As shown in Fig. 5, capacitor C charges while the LIMS is maintained in the off-state and discharges through an RLC circuit when the LIMS is triggered.

At a high temperature (125 °C), the leakage current of the prepared LIMS has been measured and is about 500 μA with a dc voltage of 5 kV (the leakage current of the traditional thyristor is larger than 1 mA). If the leakage current of the LIMS is high, part of the current for the capacitor charging is deviated by the LIMS and flows through the RLC circuit, resulting in a longer time required for charging the capacitor.

**Figure 6:**
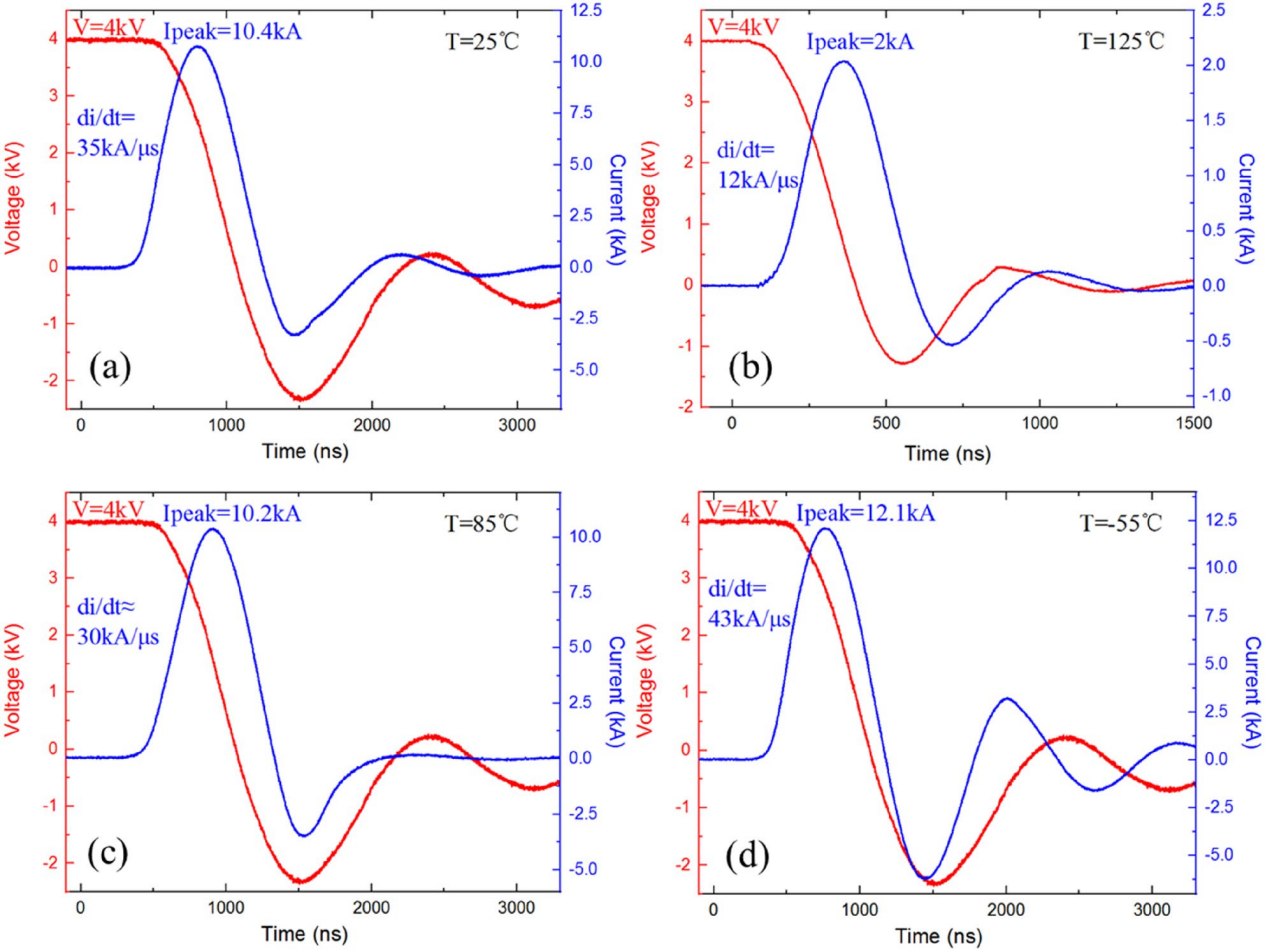
Discharging waveforms of the LIMS with a voltage of 4 kV at room temperature (**a**), at 125 °C (**b**), at 85 °C (**c**), and at − 55 °C (**d**).

Then, the turn-on characteristic of the LIMS is measured. Figure 6a presents the discharging waveforms of the LIMS with a voltage of 4 kV at room temperature. It shows that, from Fig. 6a, the peak value of the output current is about 10.4 kA and di/dt is 35 kA/μs.

Figure 6b displays the discharging waveforms of the LIMS with a voltage of 4 kV at 125 °C (here, the capacitor C in Fig. 5 is 0.1 μF). In Fig. 6b, the peak value of the output current is about 2 kA and di/dt is 12 kA/μs. Figure 6c shows the discharging waveforms of the LIMS with a voltage of 4 kV at 85 °C. In Fig. 6c, the peak value of output current is about 10.2 kA and di/dt is about 30 kA/μs.

The characteristics of the LIMS at the low temperature of − 55 °C has also been measured. The discharging waveforms are shown in Fig. 6d. The peak value of the output current is about 12.1 kA and di/dt is about 43 kA/μs.

The conclusion can be made that the prepared LIMS can work steadily at high temperatures, the leakage current is about 500 μA at 125 °C, and the output current can achieve 10.2 kA at 85 °C (di/dt is larger than 30 kA/μs).

## Conclusion

To optimize characteristics of the LIMS at high temperatures, we propose a new structure of LIMS with the optimization of the n^+^ layer, circular light gate, and the new-style edge termination, with the diameter of the LIMS being 23 mm. The experimental results show that the leakage current of the proposed LIMS has been decreased from more than 1 mA to 500 μA at 125 °C, the output current of the LIMS is 10.2 kA with a voltage of 4 kV at 85 °C, and the output current of the LIMS is 12.1 kA with a voltage of 4 kV at − 55 °C. Furthermore, di/dt is larger than 30 kA/μs. The LIMS has broad application potential in the fields of HVDC transmission or pulsed power.

## Data Availability

The data that comprise Fig.  6 are available from the corresponding author upon reasonable request.
